# Circulating miR-141 and miR-375 are associated with treatment outcome in metastatic castration resistant prostate cancer

**DOI:** 10.1038/s41598-019-57101-7

**Published:** 2020-01-14

**Authors:** A. H. Zedan, P. J. S. Osther, J. Assenholt, J. S. Madsen, T. F. Hansen

**Affiliations:** 1 Urological Research Centre, Department of Urology, Vejle Hospital, Vejle, Denmark; 2 Department of Oncology, Vejle Hospital, Vejle, Denmark; 3 Department of Biochemistry and Clinical Immunology, Vejle Hospital, Vejle, Denmark; 4 Institute of Regional Health Research, University of Southern Denmark, Odense, Denmark

**Keywords:** Prognostic markers, Prostate cancer

## Abstract

Metastatic castration resistant prostate cancer (mCRPC) is associated with high mortality, where monitoring of disease activity is still a major clinical challenge. The role of microRNAs (miRs) has been widely investigated in prostate cancer with both diagnostic and prognostic potential. The aim of this study was to investigate the relationship between circulating miRs and treatment outcome in mCRPC patients. The relative expression of five miRs (miR-93-5p, -125b-1-5p, -141-3p, -221-3p, and miR-375-3p) was investigated in plasma samples from 84 mCRPC patients; 40 patients were treated with docetaxel (DOC cohort) and 44 patients with abiraterone (ABI cohort). Blood was sampled at baseline before treatment start and at radiological progression. The plasma levels of four miRs; miR-93-5p, -141-3p, -221-3p, and miR-375-3p decreased significantly after treatment initiation in patients receiving docetaxel, and for miR-141-3p and miR-375-3p the level increased again at the time of radiological progression. In the patients treated with abiraterone, the plasma level of miR-221-3p likewise decreased significantly after the first treatment cycle. High baseline levels of both miR-141-3p and miR-375-3p were significantly associated with a shorter time to radiological progression in both cohorts. Additionally, high baseline levels of miR-141-3p and miR-221-3p were significantly associated with a shorter overall survival (OS) in the ABI cohort, while high levels of miR-141-3p and miR-375-3p were significantly associated with shorter OS in the DOC cohort. Plasma levels of miR-141-3p and miR-375-3p may predict time to progression in mCRPC patients treated with docetaxel or abiraterone. The clinical impact of these findings is dependent on validation in larger cohorts.

## Introduction

Metastatic castration resistant prostate cancer (mCRPC) is considered the most aggressive phase of the disease with survival rarely exceeding three years^1^. Virtually all advanced prostate cancer (PCa) patients will develop mCRPC within a period of about 24 months^2^.

Although the management of mCRPC has improved considerably since 2004 by the introduction of various agents providing improved overall survival (OS) and progression free survival (PFS), none of the options are curative^3^. Docetaxel and abiraterone were among the first agents to be approved in the mCRPC setting, however with a modest increase of OS^4,5^. Both agents, however, recently demonstrated a marked OS improvement in de novo metastatic PCa^6,7^.

One of the most important challenges in daily practice is the monitoring of disease activity in mCRPC patients. Prostate-specific antigen (PSA) alone is not a reliable biomarker in this stage^8^, as visceral metastases have been observed in patients without rising PSA^9^. A few prognostic models for mCRPC patients have been proposed over the last few years, but the efforts are still in their infancy^10,11^. Hence, treatment optimization at this stage of the disease may be challenged due to lack of validated non-invasive prognostic biomarkers.

MicroRNAs (miRs) are non-coding single-stranded evolutionarily conserved RNA molecules, about 22 nucleotides in length, that regulate gene expression both at the transcriptional and post transcriptional level^12^. So far, more than 2600 mature human miRs have been identified^13,14^, many with essential roles in physiological and pathological cellular processes, including cancer development and progression^15^. For different reasons, miRs have been quite attractive as alternative biomarkers, especially in PCa. Firstly, miRs have shown to be dysregulated in PCa and may be able to distinguish between stages of the disease^16^. Secondly, they can be extracted from different body fluids, including plasma^17^. Lastly, miRs remain stable under various storage conditions^18^.

The purpose of this study was to investigate the relationship between circulating miRs and treatment outcome in patients with mCRPC treated with either docetaxel or abiraterone.

## Results

### Patient characteristics

We recruited 40 patients to the DOC cohort and 44 patients in the ABI cohort.

The median PSA level at the stage of mCRPC in the DOC cohort was significantly higher than in the ABI cohort. Furthermore, the distribution of metastases differed significantly between the two cohorts. In the DOC cohort the time until mCRPC development was significantly shorter than in the ABI cohort. Due to an earlier inclusion start in the DOC cohort, the follow-up period was significantly longer compared to the ABI cohort. The clinicopathological characteristics of all patients are presented in Table 1.

**Table 1:** Clinicopathological characteristics.

	ABI cohort	DOC cohort	*p*-value
	(n = 44)	(n = 40)	
Mean age at Dx, years (range)	69 (54–84)	68 (52–81)	0.921
**Pathology at Dx**			
Adenocarcinoma	44 (100)	40 (100)	
GS at Dx			0.398
6 (3 + 3)	1	1	
7 (3 + 4)	7	8	
7 (4 + 3)	10	6	
8	13	6	
≥9	12	16	
N/A	1	3	
EAU risk group			0.715
Low	1	0	
Intermediate	5	3	
High	38	37	
Type of initial management			1.000
RARP	1	0	
RT	4	4	
Palliation ADT	39	36	
Type of castration treatment			0.856
Medical	33	32	
Surgical	4	2	
Medical then surgical	7	6	
Median PSA level at mCRPC, ng/ml (range)	36 (1–388)	68 (6–2509)	0.023
Site of metastases at mCRPC			0.048
Bone	39	38	
LN	14	17	
Liver	0	6	
Lung	1	0	
Others	1	0	
Time to mCRPC, mo, median (range)	41 (7–160)	23 (4–142)	0.025
Follow-up, mo, median (range)	35 (19–55)	64 (13–77)	<0.001
Time to RP, mo, median (range)	8 (2–23)	7 (2–30)	0.766
Death	24	30	
Synchronic cancer	7	4	

ABI: abiraterone; ADT: anti-deprivation therapy; EAU: European Association of Urology; DOC: docetaxel; Dx: Diagnosis; GS: Gleason score; LN: Lymph node; mCRPC: metastatic castration resistant prostate cancer; RARP: robot assisted radical prostatectomy; RP: radiological progression; RT: radiotherapy.

The baseline plasma levels of three microRNAs, i.e. miR-93-5p, miR-125b-1-5p and miR-221-3p, differed significantly between the two cohorts (Supplementary Table S1).

### Changes in circulating miRs during treatment

After the first treatment cycle, the plasma levels of miR-221-3p decreased significantly in both the ABI and the DOC cohort (z = −1.962, *p* = 0.049 and z = −2.168, *p* = 0.030, respectively). Furthermore, in the DOC cohort the plasma levels of miR-93-5p, −141−3p, and miR−375–3p were significantly lower than the baseline levels (z = −2.255, *p* = 0.024; z = −3.870, *p* = <0.001, and z = −4.148, *p* = <0.001 respectively) (Table 2).

**Table 2:** Association between baseline and second sample (after first treatment cycle).

	ABI cohort		DOC cohort	
	Second sample		Second sample	
	*z*-score	*p*-value	*z*-score	*p*-value
miR-93-5p	−0.680	0.496	**−2**.**255**	**0**.**024**
miR-125b-1-5p	−0.045	0.963	−0.399	0.690
miR-141-3p	−1.247	0.212	**−3**.**870**	**<0**.**001**
miR-221-3p	**−1**.**962**	**0**.**049**	**−2**.**168**	**0**.**030**
miR-375-3p	−0.505	0.613	**−4**.**148**	**<0**.**001**

ABI: abiraterone; DOC: docetaxel. Statistically significant values are in bold writing.

At progression circulating miR-141-3p and miR-375-3p increased significantly compared to the plasma levels after the first docetaxel treatment (z = 2.368, *p* = 0.018 and z = 2.146, *p* = 0.032, respectively). In the ABI cohort, the plasma level of miR-125b-1-5p at progression tended to be significantly lower compared to the level after the first abiraterone treatment (z = −1.938, *p* = 0.053) (Table 3).

**Table 3:** Association between plasma miR level in second sample and progression sample.

	ABI cohort		DOC cohort	
	Progression sample		Progression sample	
	*z*-score	*p*-value	*z*-score	*p*-value
miR-93-5p	−0.686	0.493	1.330	0.184
miR-125b-1-5p	−1.938	0.053	0.137	0.891
miR-141-3p	0.392	0.695	**2**.**368**	**0**.**018**
miR-221-3p	−1.546	0.122	1.178	0.239
miR-375-3p	−0.471	0.638	**2**.**146**	**0**.**032**

ABI: abiraterone; DOC: docetaxel. Statistically significant values are in bold writing.

**Figure 1:**
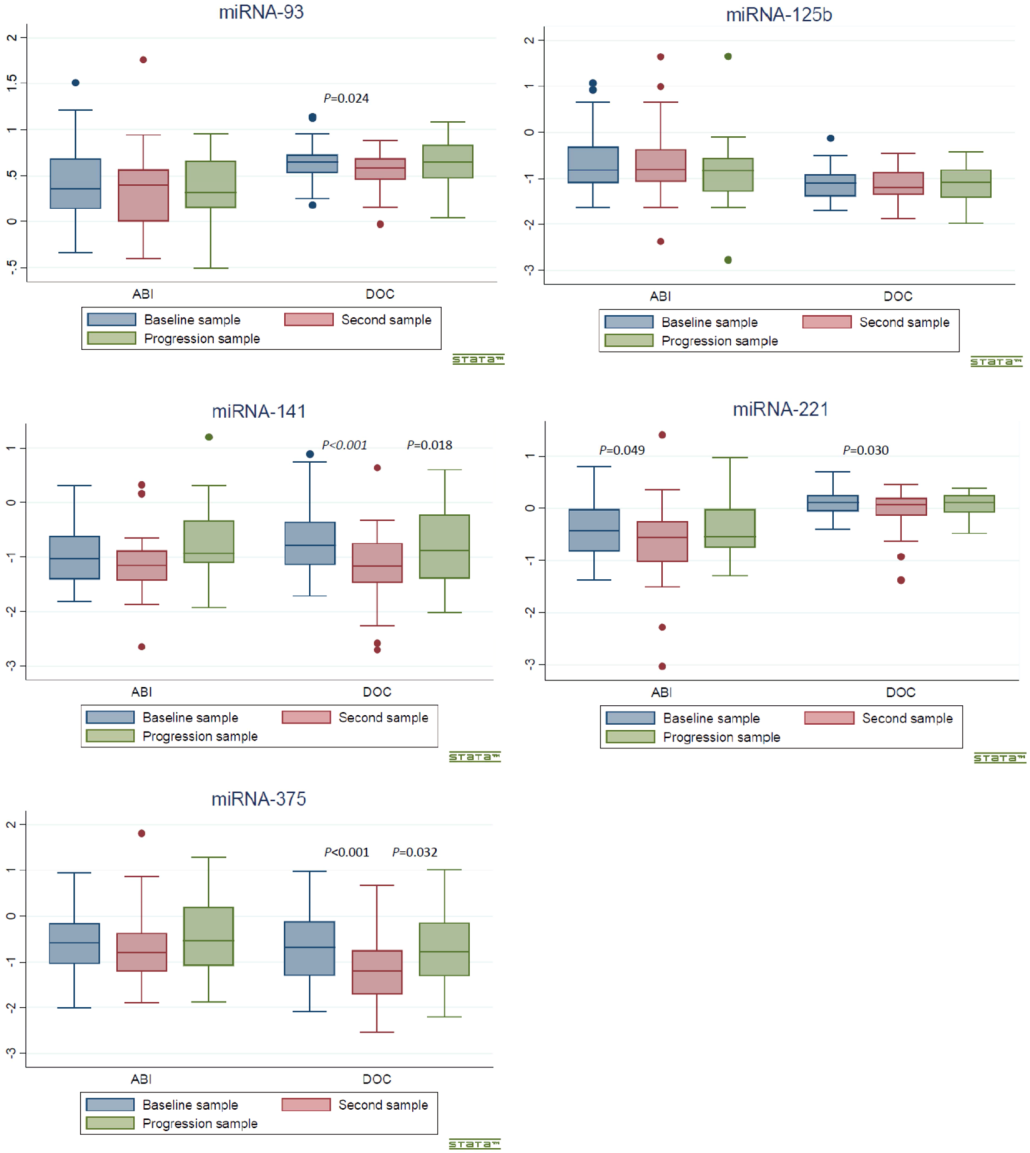
Boxplots illustrating the level of the five miRs at baseline, after one treatment cycle of treatment and at radiological progression in both the ABI and the DOC cohort. Y axis represents linear ΔCt values.

The levels of circulating miRs at baseline, after one treatment cycle, and at radiological progression in both cohorts are illustrated by boxplots in Fig. 1.

### Association between circulating miRs and treatment outcome

High plasma baseline levels of miR-141-3p and miR-375-3p were significantly associated with a shorter time to rPFS both in the ABI cohort (HR = 3.18, *p* = <0.01; HR = 2.17, *p* = 0.02, respectively), and in the DOC cohort (HR = 2.56, *p* = <0.01; HR = 2.44, *p* = <0.01, respectively).

In the ABI cohort high levels of miR-141-3p and miR-221-3p at baseline were significantly associated with shorter OS (HR = 3.20, *p* = 0.02, HR = 2.36, *p* = 0.04, respectively). In the DOC cohort, high levels of miR-141-3p and miR-375-3p were significantly associated with shorter OS (HR = 1.77, *p* = 0.02; HR = 2.48, *p* = <0.01, respectively) (Table 4).

**Table 4:** Association of plasma miRs level at baseline with rPFS and OS.

Baseline miR	ABI						DOC					
	RP			OS			RP			OS		
	HR	p-value	95% CI	HR	p-value	95% CI	HR	p-value	95% CI	HR	p-value	95% CI
miR-93-5p	2.38	0.08	0.91–6.28	1.66	0.33	0.62–4.66	1.40	0.68	0.28–6.93	0.41	0.28	0.08–1.96
miR-125b-1-5p	1.27	0.39	0.74–2.18	1.502	0.18	0.82–2.67	1.2	0.68	0.50–2.93	1.66	0.40	0.49–4.88
miR-141-3p	**3**.**18**	**<0**.**01**	**1**.**31–7**.**32**	**3**.**202**	**0**.**02**	**1**.**25–9**.**37**	**2**.**56**	**<0**.**01**	**1**.**45–4**.**50**	**1**.**77**	**0**.**02**	**1**.**07–2**.**83**
miR-221-3p	1.33	0.39	0.69–2.53	**2**.**36**	**0**.**04**	**1**.**03–5**.**34**	1.13	0.97	0.25–5.07	2.02	0.50	0.25–14.78
miR-375-3p	**2**.**17**	**0**.**02**	**1**.**14–4**.**19**	1.403	0.31	0.74–2.71	**2**.**44**	**<0**.**01**	**1**.**51–3**.**94**	**2**.**48**	**<0**.**01**	**1**.**42–3**.**67**

ABI: abiraterone; CI: confidence intervals; DOC: docetaxel; rPFS: radiological progression free survival; OS: Overall survival. Statistically significant values are marked in bold writing.

**Figure 2:**
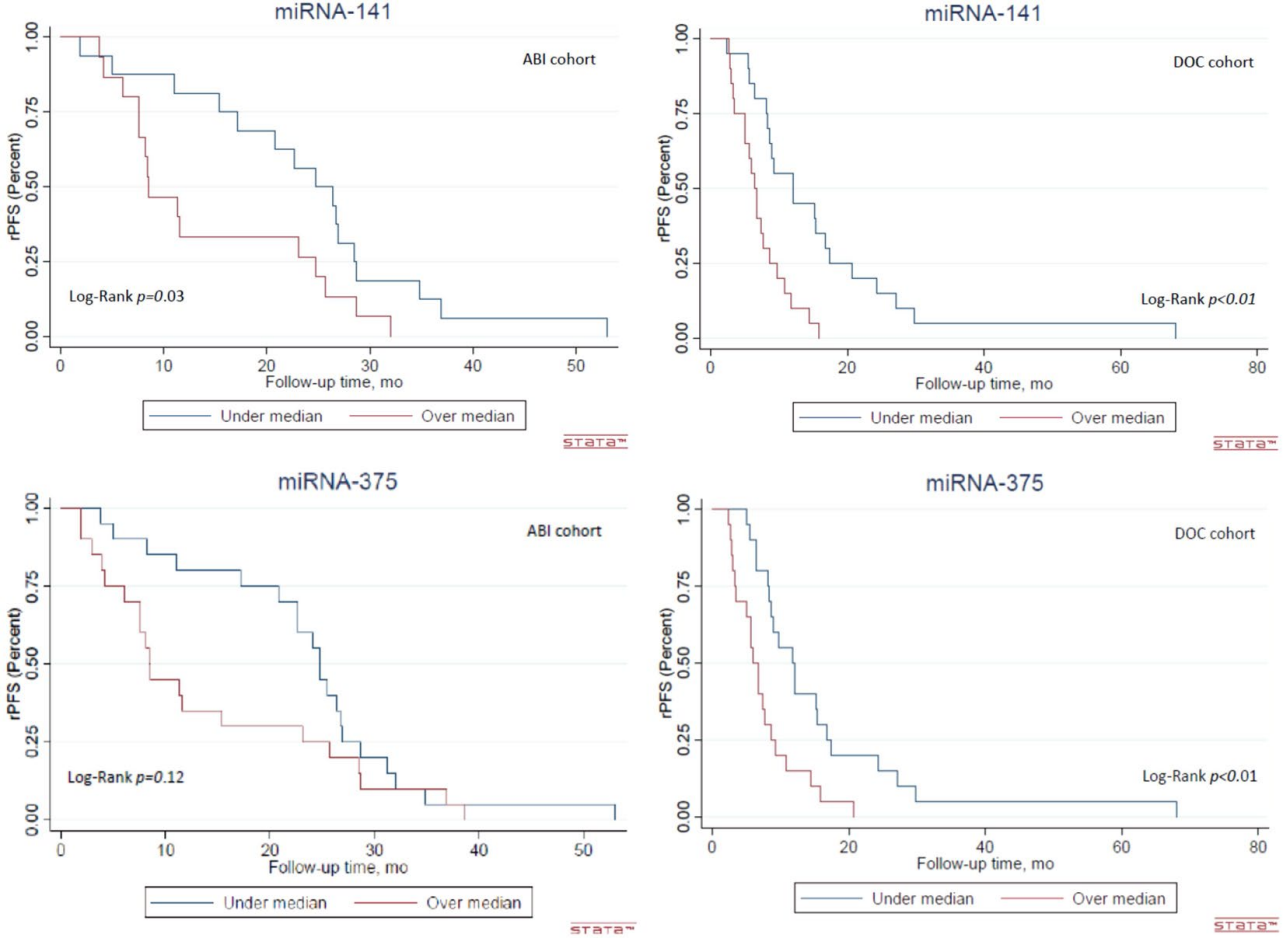
Radiological progression-free survival analysis in the DOC and the ABI cohorts for both miR-141-3p and miR-375-3p.

The association of baseline circulating miR-141-3p and miR-375-3p with rPFS is further illustrated by Kaplan-Meier curves in Fig. 2. Full OS and rPFS data are provided in Supplementary Table S2, Figs. S1 and S2.

## Discussion

In the present study we demonstrate the association of miR-141-3p and miR-375-3p with treatment outcome in mCRPC patients treated with either docetaxel or abiraterone. The level of both miRs decreased after treatment initiation and increased again at progression.

Circulating levels of miRs in mCRPC patients has been assessed in previous studies^19–21^. Plasma levels of both miR-141-3p and miR-375-3p demonstrated a relative high specificity to distinguish local PCa from mCRPC^19^. Cheng *et al*.^20^ and Nguyen *et al*.^21^ observed a higher level of a panel of miRs (including miR-141-3p and miR-375-3p) in blood samples pooled from patients diagnosed with mCRPC compared with an age-matched control group or low risk PCa patients, respectively. These results were furthermore validated in serum samples collected from another independent cohort (21 mCRPC patients versus 20 healthy controls)^20^.

The decreased plasma level of most of the investigated miRs in this study after introduction of both docetaxel and abiraterone and the subsequent increase at radiological progression argue for an oncogenic role in PCa. These changes of circulating miRs as a treatment response has also been reported in previous studies^22–24^.

Lin *et al*.^23^ documented a significant association between changes in circulating levels of six miRs (three of them are members of miR-200 family, namely miR-200b, -200c, and miR-429) and PSA response in mCRPC patients receiving docetaxel. However these results couldn’t be validated in a larger independent mCRPC cohort receiving docetaxel in a phase 2 study performed by the same group^24^. Dispite a small population size (10 mCRPC patients), a predictive value of high serum miR-21 (4/10) were proposed for those who were resistant to docetaxel treatment^22^.

Among the upregulated miRs, miR-141and miR-375 were among the first circulating miRs investigated in PCa^25,26^.

The function of both miR-141-3p and miR-375-3p in CRPC pathogenesis may be similar and interactive. SEC. 23A is a target for both miR-375-3p and the miR-200 family (miR-141-3p is one of its members), where both miRs promoted epithelial-to-mesenchymal transition (MET) in PCa^27–30^. While miR-375-3p regulates MET through YAP1, and its expression shown to be suppressed by ZEB1, miR-200 family members downregulates ZEB1 expression, and therefore may contribute to upregulation of miR-375-3p^28^.

Interestingly, the plasma levels of miR-125b-1-5p showed a tendency towards a decrease at progression in the ABI cohort. This observation is in concordance with our previous work^31^, in which the plasma level of miR-125b-1-5p decreased six months after diagnosis of local prostate cancer in a cohort of patients undergoing active surveillance. Although some studies have indicated an oncogenic role^25,32^, the exact function of miR-125b-1-5p in cancer progression is not fully understood^33^.

In the current study a higher baseline plasma level of miR-141-3p and miR-375-3p was significantly associated with shorter rPFS in mCRPC patients regardless of treatment. While higher levels of miR-141-3p and miR-221-3p at baseline were associated with poorer OS in patients receiving abiraterone, an unfavourable prognosis in the DOC cohort was characterised by high levels of miR-141-3p and miR-375-3p.

Previous studies have demonstrated a prognostic value of miR-375-3p in the mCRPC setting^21,24,34–36^. Nguyen *et al*. has proposed a prognostic value of both miR-141-3p and miR-375-3p with a trend of increasing plasma levels at disease progression from low risk PCa, high risk PCa and to mCRPC^21^. The same results were observed by Bryant *et al*. exploring both miRs in two different independent cohorts. In both plasma and sera (microvesicles and exosomes) circulating levels of both miR-141-3p and miR-375-3p were significantly correlated with metastatic PCa^35^.

Additionally, in a screening cohort of 23 mCRPC patients, high plasma level of miR-375 was associated with poor OS. This observation was validated in a follow-up cohort of another independent 100 mCRPC patients^34^.Futhermore, in a phase 2 study Lin *et al*. has observed associations between baseline levels of circulating miR-375 and poor outcome^24^.

In contrast to our findings, Kneitz *et al*.^36^ found that lower expression of miR-221-3p was associated with a higher rate of cancer related death. A possible explanation for this difference could be the patient population and material used. Kneitz *et al*. investigated the expression of miRs in paraffin-embedded prostate cancer tissue from localized PCa patients treated with radical prostatectomy.

Many studies have demonstrated a suppressed expression of miR-221-3p in PCa^37,38^. Additionally, miR-221-3p is strongly associated with aggressiveness of PCa and may serve as a potential prognostic biomarker in patients diagnosed with high-risk PCa^39^. Interestingly, other cancers such as lung, bladder, thyroid, breast, liver, or pancreas showed an overexpression of miR-221-3p where the tumor suppressor p57kip2, c-kit, PTEN TIMP3, and PUMA have been reported to be miR-221 targets^40–43^.

We observed a relative more missed/over 37 Ct values in plasma samples after treatment initiation compared with baseline samples. This was more obvious for miR-141-3p, miR-221-3p and miR-375-3p. Such observations were not unexpected, because treatment with either abiraterone or docetaxel would probably influence the amount of detectable oncogenic miRs as it was hypothesized in our study. However, there was a decrease of these missed/over 37 Ct values at time of prostate cancer progression (due to increase in plasma level of these two oncogenic miRs), especially for both miR-141-3p and miR-221-3p in ABI cohort. This could be explained by the different mechanism of both abiraterone and docetaxel. Abiraterone is considered as a new agents to modulate androgen production, and regulating effect of both miR-141-3p and miR-221-3p on androgen receptor transcription has been documented^44,45^.

It is worth noting that the mCRPC patients in the DOC cohort had a more aggressive disease compared to the patients in the ABI cohort. This could be observed from the relatively high PSA level and higher incidence of visceral metastases in the DOC cohort. The site of metastases has been suggested to correlate with prognosis, visceral metastases having the worst outcome^46^.

The serial blood sample collection from two different cohorts and the long follow-up period are considered strengths in the present study. The solidity of our findings has also been emphasized despite the heterogeneity of the patients and different treatments in the cohorts. However, the small size of the DOC and the ABI cohorts could be considered a limitation, therefore external validation of these results is required as the purpose of the current study was explorative and hypothesis generating.

Larger cohorts are required to validate the above results. Additionally, investigation of the treatment effect of other options such as cabazitaxel, enzalutamide or radium-223 could explore more about the significance of miRs in the mCRPC setting.

Heterogeneity of mCRPC is a well-established clinical challenge, and patient stratification based on metastatic tissue biopsies is not routinely applicable. In a time with many treatment options robust, non-invasive biomarkers guiding the choice of the most appropriate treatment are warranted.

## Conclusion

The plasma level of miR-141-3p and miR-375-3p in mCRPC patients decreases after treatment initiation of abiraterone as well as docetaxel and increases again at the time of radiological progression. Both miRs demonstrated a significant relationship with treatment outcome and overall survival in both cohorts. These results add to the clinical significance of miRs as alternative and non-invasive biomarkers in prostate cancer. Validation of our results in larger cohorts will help clarify the clinical impact.

## Materials and Methods

### Patients

This study was based on plasma samples prospectively collected from two cohorts of mCRPCa patients; the docetaxel (DOC) cohort, and the abiraterone (ABI) cohort.

The patients were enrolled at the Department of Oncology, Vejle Hospital, Denmark. In total, 84 mCRPCa patients were recruited; 40 patients in the DOC cohort from June 2012 to August 2017 and 44 in the ABI cohort from April 2014 to March 2017. All participants provided informed written consent.

Seven patients in the ABI cohort had a synchronic cancer (two with colon cancer, one with lung cancer, one with testicular and liver cancer, one with malignant melanoma, one with urothelial cancer, and with thyroid cancer). Four patients in the DOC cohort had a synchronic cancer (two patients with adenocarcinomas of the skin, one with Hodgkin’s lymphoma and one patient with renal cancer).

Detailed clinicopathological data for all patients in both cohorts is provided in Supplementary Table S3.

The study was approved by The Regional Committees on Health Research Ethics for Southern Denmark (S-20110102 and S-20130099 for DOC and ABI protocol, respectively) and The Danish Data Protection Agency according to Danish law. The Danish Registry of Tissue Utilization was screened prior to study initiation. All methods were performed in accordance with the relevant guidelines and regulations.

Patients in the DOC cohort were treated with a maximum of eight cycles of docetaxel, 75 mg/m^2^ every three weeks, unless radiologic progression or unacceptable toxicity occurred earlier. The ABI cohort was treated with abiraterone tablets 1000 mg + prednisone tablets 10 mg daily until radiological progression or unacceptable toxicity.

All patients were evaluated clinically and biochemically before every treatment cycle (every 3 weeks in the DOC cohort and every 4 weeks in the ABI cohort), and radiologically by bone scan as well as chest and abdomen computed tomography (CT) every 3 cycles.

For the analysis of OS a clinical cut-off date was set for September 27, 2018. The follow-up period was defined as the time from inclusion to the clinical cut-off date. Time to mCRPC was calculated from the date of PCa diagnosis to the date of verified mCRPC. The definition of mCRPC was based on the European Association of Urology (EAU) guidelines^47^.

Overall survival was defined as the time from treatment start to death from any cause and radiologic progression free survival (rPFS) as the time from treatment start to radiological progression or death, whichever occured first. Radiological progression was assessed on the basis of the Prostate Cancer Working Group 3 (PCWG3) criteria^48^, while soft-tissue disease progression was evaluated by modified Response Evaluation Criteria In Solid Tumors (RECIST) 1.1^49^. Progression of bone metastases was evaluated by bone scans with two or more new lesions indicating progression.

At clinical cut-off date there were 19 patients in the ABI cohort and 24 patients in the DOC cohort who had radiological progression.

### Blood collection and storage

Three plasma samples from each patient in both cohorts were chosen for further analysis; at baseline, just after treatment initiation, and at radiological progression.

Venous blood samples were collected from all patients in both cohorts at baseline and radiological progression. Sampling was performed by skilled phlebotomists using a minimum of venous stasis to prevent hemolysis. Whole blood was collected into 9 mL ethylenediaminetetraacetic acid (EDTA) containing tubes (Becton-Dickinson, Franklin Lakes, NJ, USA). The samples were centrifuged at 2500 g for 10 min. and carefully transferred into cryo-tubes leaving approximately 1 mL of plasma on top of the buffy coat and then stored at −80 °C. A second step of centrifugation at 3000 g for 30 min. was carried out after storage but prior to miR purification.

To reduce the risk of hemolysis and release of miRs from other intravascular cell compartments blood was centrifuged within 30 minutes after sampling. Also, all samples were evaluated for hemolysis by A414 measure, and approx. 92% of all values were ≤log (abs) = 0.2, which corresponds to less than 1% hemolysis. There were 21 samples (8%) above 0.2 and below 0.5. Evaluation of them did not show any trends towards neither higher nor lower cycle threshold (Ct) values and therefore, they were not excluded. Only one sample (0.4%) had a relatively high hemolysis index (≥0.5) and this sample was excluded from further data analysis.

#### Selected miR targets

In the present study a panel of five miRs was selected; miR-93-5p, -125b-1-5p, -141-3p, -221-3p, and miR-375-3p. This choice was based on results from our previous work^31,50,51^ and a comprehensive review of the literature.

### RNA preparation and qPCR

#### Plasma samples

miR purification of plasma samples was performed using the Maxwell RSC miR Tissue Kit (AS1460) verified for plasma use (Scientific Style and Format, 7^th^ edition, 2006). 200 µl plasma was mixed with 200 µl chilled 1-Thioglycerol/Homogenization solution and 200 µl lysis solution. After incubation for 10 min. the sample mix was loaded into the Maxwell RSC Instrument. Elution was done using 60 µl elution buffer.

#### Reverse transcription and qPCR

All preparations were done using the standard protocol from the QIAGEN miRCURY™ LNA™ miR PCR System. Universal cDNA synthesis was performed in duplicate using miRCURY™ LNA™ miR RT Kit (QIAGEN Cat. No. 339340) in 10ul reaction volume using 2 ul purified RNA in each reaction. cDNA was then diluted 1:40 in water for qPCR performed in 10 ul reaction volume using miRCURY™ LNA miR Custom PCR panels (Cat. No 339330) and miRCURY™ SYBR Green Master Mix (Cat. No. 339345) supplied by QIAGEN.In brief, we used a plate setup supplied by QIAGEN with all primers as dried pellets in individual plate wells. After universal RT the individual miR’s are detected using LNA-enhanced miR species specific primers and SYBR green. We used Roche Lightcycler 96®.

### Use of controls

Each patient sample was added several exogenous controls. First, before purification, UniSp2/4/6 template mix was spiked in to assess RNA purification efficiency. Next, Cel-miR-39 template was spiked in before RT reaction to assess the level of inhibition in this reaction. Plate-to-plate variation was eliminated using 6× UniSp3 IPC template on each plate (each plate was commercially supplied with both template and primers for UniSp3). UniSp4 and Cel-miR-39 added templates were then amplified in qPCR using target specific LNA primers. This control setup was part of the miRCURY™ LNA™ miR PCR System and we followed the general guideline for this protocol regarding volumes and concentrations, ie. we added 0,02 fmol UniSp4 to each purification reaction and 0,001 fmol Cel-miR-39 to each RT reaction. In addition, on each plate we tested all miR assays using “no template controls”. For normalization of miR levels, we used miR-17-5p and miR-191-5p, as these species had previously been established as suitable endogenous controls^51^.

### Initial data analysis

Each Ct value presented in this work is a mean Ct calculated from two qPCR results based on two individual RT reactions using RNA from the same purification. All Plate-to-plate variation controls showed very little variation (Ct mean of 20,31 and varied between plates under 0,2 Ct) for all 71 plates. The added Cel-miR-39 template was detected in all sample preparations and resulted in a mean Ct of 25.8 and a variance of 1.07 Ct. The added UniSp4 template was detected in 264 of 273 samples with a mean Ct of 31.8 and a variance of 5.4 Ct, which we conclude is more than optimal, but explainable by the sub optimal initial sample preparation. A dataset with all raw Ct values is provided in Supplementary Table S4.

### Statistical analysis

Data are presented as means of two technical replicates per plasma sample from each patient. Based on the Ct value, the fold change of plasma miR was calculated according to the following formula:

$$\Delta {\rm{Ct}}={\rm{mean}}\,{\rm{Ct}}\,{\rm{value}}\,{\rm{of}}\,{\rm{target}}\,{\rm{miR}}-{\rm{mean}}\,{\rm{Ct}}\,{\rm{value}}\,{\rm{of}}\,{\rm{miR}}-17\,{\rm{and}}\,{\rm{miR}}-191.$$

Results were linearized using 2^−(ΔCT)^, which was subsequently applied in the statistical analysis.

Samples with missed Ct values were dismissed. Likewise, Ct values above 37 measurements were excluded; however, this was not applied on samples collected after the first treatment cycle, as a decrease in miR plasma levels was expected (Supplementary Table S5a,b).

Wilcoxon rank sum test was applied for assessment of the difference in baseline levels between the DOC and ABI cohorts. The Wilcoxon signed rank sum test was used to analyse the difference between plasma miR levels at baseline, after one treatment cycle, and at progression in both the ABI and the DOC cohort and illustrated by boxplots.

The association between miR levels and OS and rPFS was assessed using simple Cox regression (univariate analysis) and Kaplan-Meier survival curves. Proportional hazard assumption for the Cox regression model was assessed by Schoenfeldt residuals, and log-Rank was used to test for differences between Kaplan-Meier survival curves.

All analyses were performed in Stata version 15.1 (StataCorp LLC, TX, USA), and correlations/differences were considered statistically significant with a p-value was < 0.05.

## Supplementary information

Supplementary figures S1 and S2.

Supplementary Dataset 1.

Supplementary Dataset 2.

Supplementary Dataset 3.

Supplementary Dataset 4.

Supplementary information 5a

Supplementary information 5b
