# Supplementary material for: Circulating miR-141 and miR-375 are associated with treatment outcome in metastatic castration resistant prostate cancer
Source: Sci Rep. 2020 Jan 14;10:227. doi: 10.1038/s41598-019-57101-7 (PMC6959345; doi:10.1038/s41598-019-57101-7)
Supplement: Supplementary file 1 — Supplementary figures S1 and S2. [file 41598_2019_57101_MOESM1_ESM.pdf]

Circulating miR-141 and miR-375 are associated with treatment outcome in metastatic castration resistant prostate cancer

A. H. Zedan, P. J. S. Osther, J. Assenholt, J. S. Madsen, T. F. Hansen

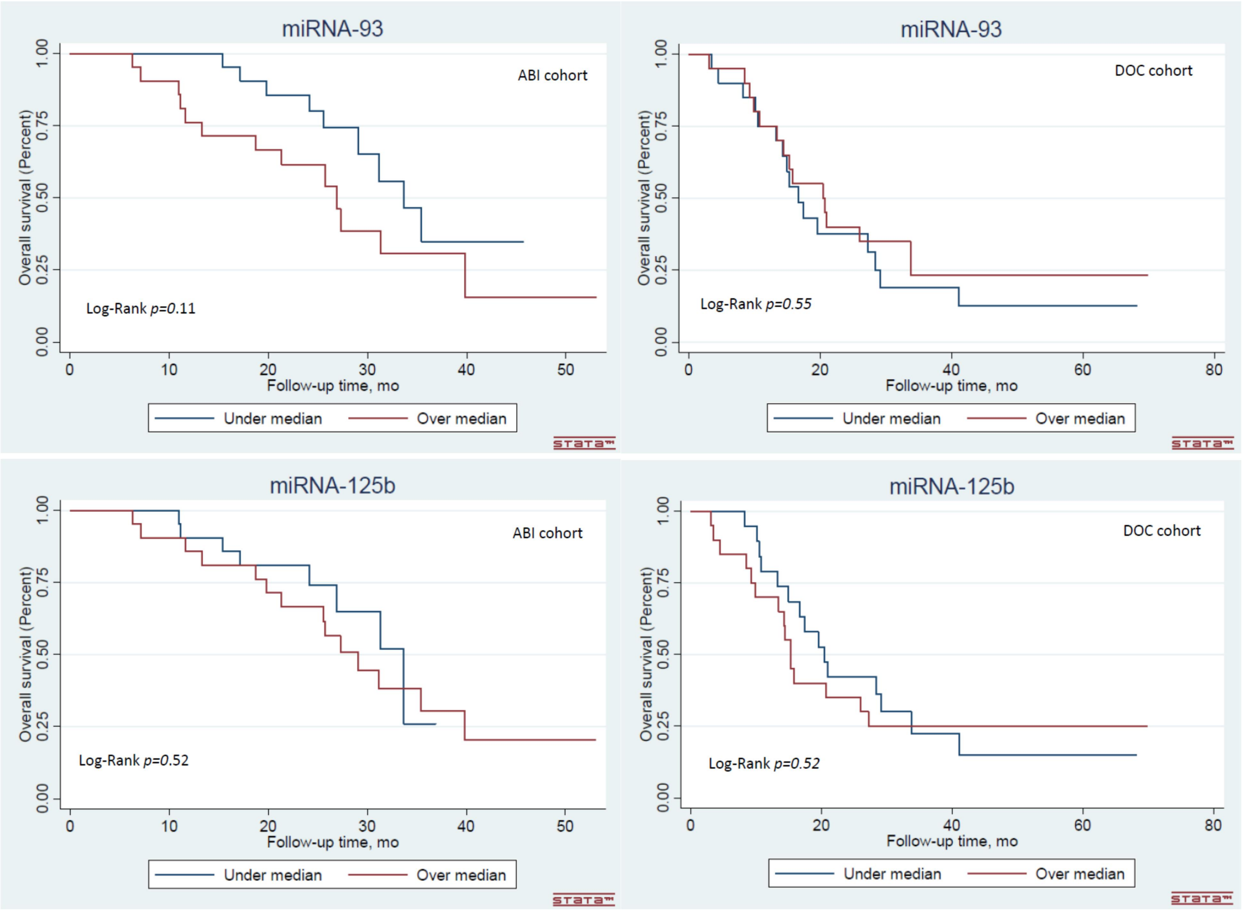
miRNA-93
ABI cohort
Log-Rank p=0.11
Overall survival (Percent)
Follow-up time, mo
Under median
Over median
miRNA-93
DOC cohort
Log-Rank p=0.55
Overall survival (Percent)
Follow-up time, mo
Under median
Over median
miRNA-125b
ABI cohort
Log-Rank p=0.52
Overall survival (Percent)
Follow-up time, mo
Under median
Over median
miRNA-125b
DOC cohort
Log-Rank p=0.52
Overall survival (Percent)
Follow-up time, mo
Under median
Over median

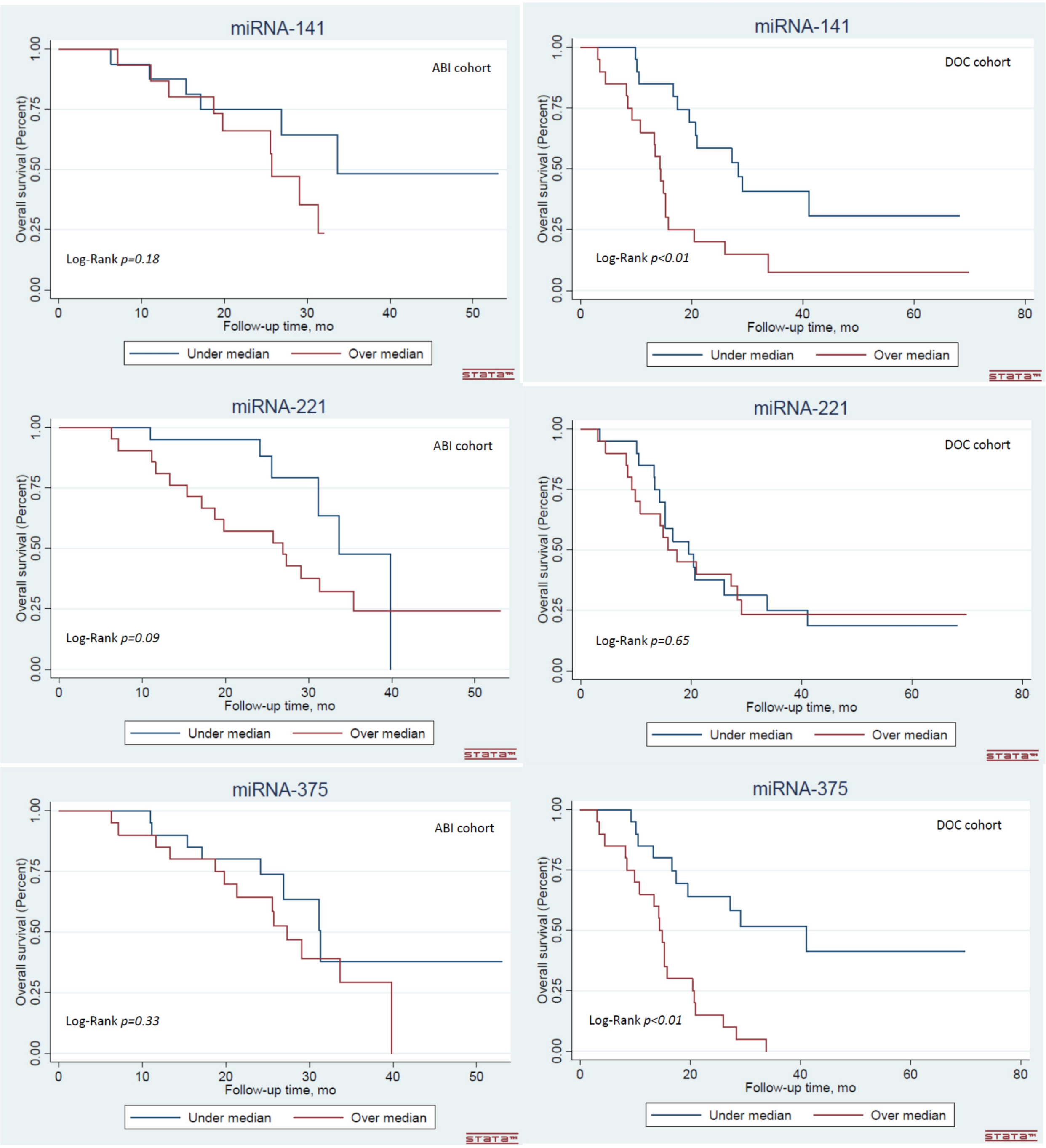

Figure S1: miR-based overall survival analysis in the abiraterone (ABI) and docetaxel (DOC) cohorts

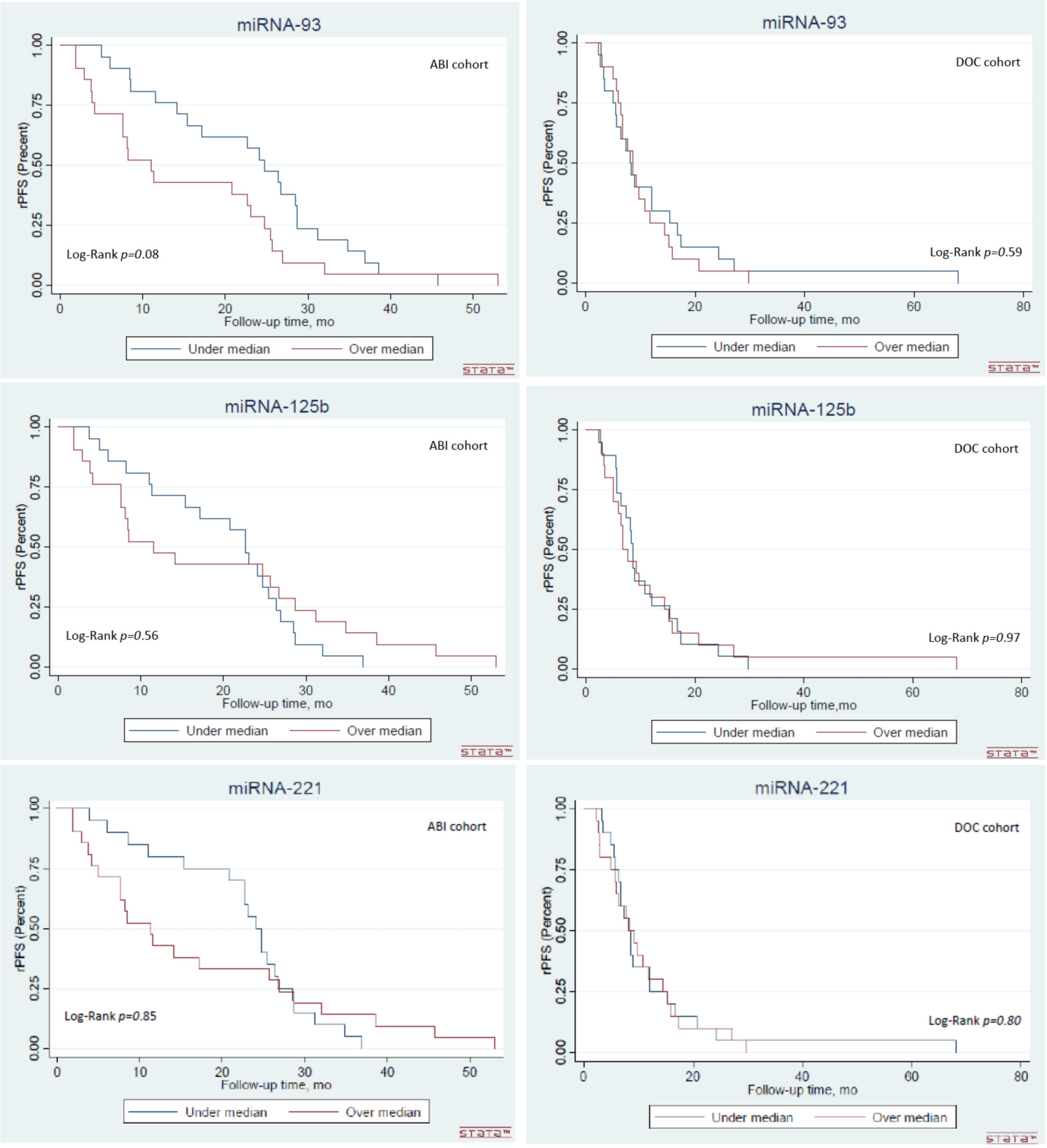

Figure S2: miR-based radiological progression-free survival analysis in the abiraterone (ABI) and docetaxel (DOC) cohorts
