# Supplementary material for: Circulating miR-141 and miR-375 are associated with treatment outcome in metastatic castration resistant prostate cancer
Source: Sci Rep. 2020 Jan 14;10:227. doi: 10.1038/s41598-019-57101-7 (PMC6959345; doi:10.1038/s41598-019-57101-7)
Supplement: Supplementary file 2 — Supplementary Dataset 1. [file 41598_2019_57101_MOESM2_ESM.pdf]

Circulating miR-141 and miR-375 are associated with treatment outcome in metastatic castration resistant prostate cancer

A. H. Zedan, P. J. S. Osther, J. Assenholt, J. S. Madsen, T. F. Hansen

Table S1: Difference in baseline plasma miRs between ABI and DOC cohort

Baseline miR	Z-score	p-value
miR-93-5p	**-3,45**	**<0.01**
miR-125b-1-5p	**3.66**	**<0.01**
miR-141-3p	-1,81	0.07
miR-375-3p	0.50	0,62
miR-221-3p	**-4,87**	**<0.01**

ABI: abiraterone; DOC: docetaxel. Statistically significant values are in bold writing
