# Supplementary material for: Circulating miR-141 and miR-375 are associated with treatment outcome in metastatic castration resistant prostate cancer
Source: Sci Rep. 2020 Jan 14;10:227. doi: 10.1038/s41598-019-57101-7 (PMC6959345; doi:10.1038/s41598-019-57101-7)
Supplement: Supplementary file 3 — Supplementary Dataset 2. [file 41598_2019_57101_MOESM3_ESM.pdf]

Circulating miR-141 and miR-375 are associated with treatment outcome in metastatic castration resistant prostate cancer

A. H. Zedan, P. J. S. Osther, J. Assenholt, J. S. Madsen, T. F. Hanse

Table S2: The association of baseline circulating miRs with OS and rPFS as demonstrated by Kaplan-Meier survival curves

	ABI cohort		DOC cohort	
	rPFS	OS	rPFS	OS
	p-value	*p*-value	*p*-value	*p*-value
miR-93-5p	0.08	0.11	0.59	0.55
miR-125b-1-5p	0.56	0.52	0.97	0.52
miR-141-3p	**0.03**	0.18	**<0.01**	**<0.01**
miR-375-3p	0.12	0.33	**<0.01**	**<0.01**
miR-221-3p	0.85	0.09	0.801	0.65

ABI: abiraterone; DOC: docetaxel. Statistically significant values are in bold writing
