# Supplementary material for: Circulating miR-141 and miR-375 are associated with treatment outcome in metastatic castration resistant prostate cancer
Source: Sci Rep. 2020 Jan 14;10:227. doi: 10.1038/s41598-019-57101-7 (PMC6959345; doi:10.1038/s41598-019-57101-7)
Supplement: Supplementary file 6 — Supplementary information 5a [file 41598_2019_57101_MOESM6_ESM.pdf]

Circulating miR-141 and miR-375 are associated with treatment outcome in metastatic castration resistant prostate cancer

A. H. Zedan, P. J. S. Osther, J. Assenholt, J. S. Madsen, T. F. Hansen

Table S5a: Missed Ct values and Ct values above 37 in the ABI and DOC cohorts

		ABI cohort			DOC cohort		
		Baseline	After 1 cycle	Progression	Baseline	After 1 cycle	Progression
**miR-93-5p**	Missed value	0	1	0	0	1	0
	Values over 37	0	2	3	0	0	1
**miR-125b-1-5p**	Missed value	1	1	0	1	0	2
	Values over 37	5	7	6	4	2	0
**miR-141-3p**	Missed value	13	15	5	0	3	1
	Values over 37	1	3	1	2	2	0
**miR-221-3p**	Missed value	1	2	1	0	2	1
	Values over 37	3	8	1	0	0	0
**miR-375-3p**	Missed value	2	3	5	0	3	2
	Values over 37	5	4	3	2	6	3

ABI: abiraterone; DOC: docetaxel
